# Volatile Organic Compounds of *Wickerhamomyces anomalus* Prevent Postharvest Black Spot Disease in Tomato

**DOI:** 10.3390/foods13121949

**Published:** 2024-06-20

**Authors:** Xi Zhang, Qiya Yang, Dhanasekaran Solairaj, Nashwa M. A. Sallam, Marui Zhu, Shengyu You, Hongyin Zhang

**Affiliations:** 1 School of Food and Biological Engineering, Jiangsu University, Zhenjiang 212013, China; xi15612806202@163.com (X.Z.); yangqiya1118@163.com (Q.Y.); solaibt@hotmail.com (D.S.); zhumarui@126.com (M.Z.); a2352296226@126.com (S.Y.); 2 Department of Plant Pathology, Faculty of Agriculture, Assiut University, Assiut 71526, Egypt; nashwasallam@aun.edu.eg

**Keywords:** tomato, *Alternaria alternata*, *Wickerhamomyces anomalus*, isoamyl acetate, GC-MS

## Abstract

Postharvest diseases, such as black spots caused by *Alternaria alternata*, have caused huge economic losses to the tomato industry and seriously restricted its development. In recent years, biological control has become a new method to control postharvest diseases of fruits and vegetables. Our research group screened *W. anomalus*, a yeast demonstrating a promising control effect on a postharvest black spot disease of tomatoes, and explored its physiological mechanism of prevention and control. Therefore, this study investigated the prevention and control effect of metabolites of *W. anomalus* on tomato black spot disease and the inhibition effect of main components on *A. alternata*. A GC-MS analysis found that isoamyl acetate was the main component of *W. anomalus* that played an inhibitory role. The results showed that isoamyl acetate could inhibit the growth of *A. alternata* and had a certain control effect on postharvest black spots in tomatoes. Our findings suggest that isoamyl acetate could be a promising alternative to fungicides for controlling postharvest black spots in tomatoes.

## 1. Introduction

The tomato (*Solanum lycopersicon* L.) is an annual herb of the genus *Solanum*, rich in carotene, vitamin B, vitamin C, and other nutrients. Still, it is easy to cause mechanical damage or be infected by pathogens during postharvest transportation and storage. *Alternaria alternata* is a common pathogen that can infect many kinds of fruits and vegetables, particularly the yield of tomatoes, and bring substantial economic loss to the fruit and vegetable industry worldwide [1]. At present, the common methods to control postharvest diseases of fruits and vegetables mainly include physical, chemical, and biological methods. Due to the high cost and difficult operation of physical methods and the long-term use of chemical fungicides poses human body and environmental risks, the green and efficient characteristics of the biological control method make it the best choice for preventing and controlling postharvest diseases of fruits and vegetables. The biological control method controls pathogen growth by introducing safe and harmless antagonistic microorganisms based on the principle of mutual antagonism between microorganisms [2]. Compared with other antagonistic microorganisms, antagonistic yeast has advantages, such as a more substantial antagonistic effect [3], no production of mycotoxins and antibiotics, genetic stability, strong environmental adaptability, and environmental friendliness [4]. Some antagonistic microorganisms can not only control postharvest diseases of fruits and vegetables but also degrade the toxins produced by pathogenic microorganisms [5], thus becoming a new method for controlling postharvest diseases of fruits and vegetables.

Antagonistic yeast can produce secondary metabolites with antimicrobial activity, and the active metabolites mainly include toxin protein, volatile organic compounds (VOCs), and so on. The cell-free extracts from *Cryptococcus albidus* KKUY0017 and *Wickerhamomyces anomalus* KKUY0051 have been found to inhibit *Penicillium expansum*. Gas chromatography–mass spectrometry revealed that the cell-free extract of these kinds of yeasts contains toxic compounds, such as nitrophenol derivatives, and further verified the inhibitory effect of this compound on pathogenic bacteria [6]. Many yeasts with distinctive odors are often considered major emitters of VOCs, and the VOCs pose antifungal efficiency. VOCs are low molecular weight metabolites with low polarity and high vapor pressure [7]. VOCs are mainly composed of alcohols, aldehydes, esters, aromatic hydrocarbons, aliphatic hydrocarbons, nitrites, and sulfides and have an inhibitory effect on postharvest pathogens under closed conditions [8].

In recent years, VOCs produced by microorganisms have attracted more and more attention as biocontrol fumigants and relevant studies have shown that their volatiles can inhibit a variety of postharvest pathogens in fruits and vegetables. Previous studies have found that VOCs of *Saccharomyces cerevisiae* can inhibit filamentous fungi, bio-fumigate citrus, and control postharvest blight [9]. VOCs are produced in different metabolic processes and can be divided into different categories, such as alcohols, esters, acids, aldehydes, ketones, etc. Common alcohol volatiles include ethanol, 3-methyl-butanol, phenyl ethanol, etc.; esters mainly include ethyl acetate, butyl 3-methylacetate, isoamyl acetate, etc.; and acid substances mainly include acetic acid, capric acid, etc. [10]. Volatile substances do not require direct contact with food, which eliminates consumers’ concerns about biological residues. It is suitable for fumigation control of various fruits and vegetables. Therefore, it is of great significance to explore the antifungal mechanism of microbial volatile substances for commercial applications [11]. Relevant experimental research has discovered that volatile compounds inhibit pathogenic bacteria through a mechanism. This involves damaging the structure of the pathogenic bacteria’s cell wall membrane, preventing the spore germination and sprout tube length of the pathogenic bacteria [12], and subjecting the pathogenic bacteria to oxidative stress [13]. According to Contarino et al., the majority of antagonistic yeasts exhibit unique species-specific VOC production capabilities as well as specific antibacterial effects on a variety of pathogens. That could help avoid and manage fruit and vegetable postharvest diseases [14]. The report and research of antifungal VOCs produced by antagonistic yeast are in the initial stage, and their biocontrol effects on postharvest diseases of fruits and vegetables need to be further studied. In the process of the postharvest storage and transportation of fruits and vegetables, the use of VOC-producing microorganisms as biological fumigants is expected to be an alternative method to control postharvest diseases of fruits and vegetables [13]. The fumigation of fruits and vegetables by the volatile metabolites of antagonistic microorganisms is a green and environmentally friendly way, which provides a theoretical basis for disease control in fruits and vegetables in the future.

Previous studies in our research group found that *W. anomalus* could induce host resistance to oxidative stress, improve the activity of antioxidant-related enzymes, and strengthen the activity of tomato stress-related enzymes, thus improving host resistance. *W. anomalus* can inhibit the growth and development, spore germination, and bud tube elongation of *A. alternata* when infected by pathogens; compete for nutrients and space in tomatoes; and play an important role in preventing and controlling postharvest black spots in tomatoes [15]. However, the biocontrol effects of the metabolites produced by *W. anomalus* have not been studied. Therefore, in order to deeply explore the mechanism of *W. anomalus* prevention and the control of postharvest black spots in tomatoes, this study conducted analyses on its biological control efficacy and key components. This included examining its nonvolatile and volatile metabolites’ effects on the growth of *A. alternata* and tomato black spot disease, conducting a GC-MS analysis of *W. anomalus* VOCs, and assessing the impact of isoamyl acetate fumigation on *A. alternata* in vitro and on tomato black spot disease. The research provides a theoretical reference for controlling postharvest diseases of fruits and vegetables by using antagonistic yeast fumigation.

## 2. Materials and Methods

### 2.1. Yeast and Pathogen

*W. anomalus* was activated in nutrient yeast dextrose broth (NYDB) medium. After 20 h (28 °C, 180 rpm) incubation (ZQLY-180N, Shanghai Zhichu Instrument, Shanghai, China), the activated yeast was inoculated in a fresh medium and cultured for 20 h (28 °C, 180 rpm). The yeast culture was centrifuged (5810R, Eppendorf, Hamburg, Germany) (20 °C, 9600× *g*) for 5 min and washed with normal saline twice to obtain yeast precipitation. The initial concentration was counted with a blood cell counter and adjusted to 1 × 10^8^ cells/mL and used further. *A. alternata* used in this experiment was isolated from diseased tomato fruits caused by black spot disease. The pathogenic isolate was identified with molecular techniques and stored in a refrigerator at −80 °C. Spore suspension of 1 × 10^5^ spores/mL was prepared.

### 2.2. Assessing of Nonvolatile Metabolites of W. anomalus on A. alternata

After inoculating *W. anomalus* (1%) in NYDB medium, the yeast was cultured for 1 d and 5 d (28 °C, 180 rpm), then centrifuged using a high-speed refrigerated centrifuge (12,000× *g*, 4 °C) for 10 min. The supernatant was filtered with a sterile organic filter membrane (0.22 μm) to obtain cell-free supernatant. The heat-killed cell-free supernatant was obtained with high-temperature sterilization (121 °C, 15 min).

The potato dextrose agar (PDA) solid medium was center-punched with 8 mm diameter holes, and 20 μL of cell-free supernatant (Y1 and Y5, supernatant collected from 1 and 5 day culture, respectively), heat-killed cell-free supernatant (Y1-H and Y5-H, supernatant collected from 1 and 5 day heat-killed culture, respectively), and sterile water (CK) was added, respectively. The *A. alternata* spore suspension of 20 μL was added into the hole at an interval of 10 min and cultured continuously for 7 days. The growth trend of pathogen was observed, and the growth diameter was measured. The experiment was repeated twice with 3 parallel subjects in each group [16]. Details about the control and treatment groups of this experiment are listed in Table S1.

### 2.3. Impact of Volatile Metabolites of W. anomalus on the Growth of A. alternata

Solid media NYDA, PDA, and yeast extract peptone dextrose medium (YPD) were covered with 100 μL yeast suspension (1 × 10^8^ cells/mL) for 24 and 48 h, respectively. In the control groups, sterile saline was added to the solid media instead of yeast suspension. Details about the specific control and treatment groups are provided in Table S2. The center of solid medium was drilled, and 20 μL fungal spore suspension was added, dried, interlocked with yeast plates cultured for 48 h, wrapped with a sealing film, and cultured continuously for 7 days at 25 °C. The growth state of *A. alternata* was recorded every day [16].

### 2.4. Effect of W. anomalus Volatile Substances on Tomato Black Spot Disease

A sterile sealed box was filled with 350 mL of PDA medium. For the treatment group (Y), 1 mL of *W. anomalus* was inoculated into the medium. The control group (CK) was added with sterile water instead of yeast. Tomatoes inoculated with *A. alternata* were placed in a sterile sealed box and stored under a constant temperature seal (20 °C). The black spot incidence of tomatoes was observed 5 days later [16]. Details about the control and treatment groups of this experiment are listed in Table S3.

### 2.5. GC-MS Analysis of W. anomalus VOCs

The vial was filled with 3 mL of PDA medium, 10 μL *W. anomalus* was inoculated, and sterile water was used as the control group. After culturing for 24 h, the main components of volatile substances were detected and analyzed by gas chromatography–mass spectrometry (GC-MS) (GCMS-TQ8040, SHIMADZU, Izumo, Shimane Prefecture, Japan) [16]. The mass spectrometry results were automatically retrieved and substances with a matching degree greater than 80 were retained. Volatile compounds not found in the control group were used as components of *W. anomalus* volatile substances, and the relative contents of each compound were calculated.

### 2.6. Effect of Isoamyl Acetate Fumigation on A. alternata In Vitro

Two sterile filter paper sheets were positioned at 1/3 and 2/3 of the PDA plate’s center line. On one of the filter paper sheets, 10 μL of *A. alternata* (1 × 10^5^ spores/mL) were dripped, and on the other filter paper sheet, 10 μL of isoamyl acetate were dripped (purity ≥ 97%) (I112108, Aladdin, Los Angeles, CA, USA). Plates without fumigation served as the control group (CK). The plate was sealed, and the growth state of *A. alternata* was continuously observed with static culture (25 °C) [16]. Details about the control and treatment groups of this experiment are listed in Table S4.

### 2.7. Influence of Isoamyl Acetate Fumigation on Tomato Postharvest Black Spot Disease

Four pieces of filter paper were added into the sterile sealed box, and each filter paper was injected with 500 μL isoamyl acetate. The tomato inoculated with *A. alternata* was placed in the sterile sealed box for constant temperature sealed storage (20 °C). Filter paper was injected with 500 μL of sterile water and served as control group. The black spot incidence of the tomato was observed after 5 days [16]. Details about the control and treatment groups of this experiment are listed in Table S5.

### 2.8. Statistical Analysis

Data were analyzed using Excel 2021 and IBM SPSS Statistics version 26, and *t*-test was performed. *p* < 0.05 was considered as significant difference.

## 3. Results

### 3.1. Effect of Nonvolatile Metabolites of W. anomaluson A. alternata

**Figure 1:**
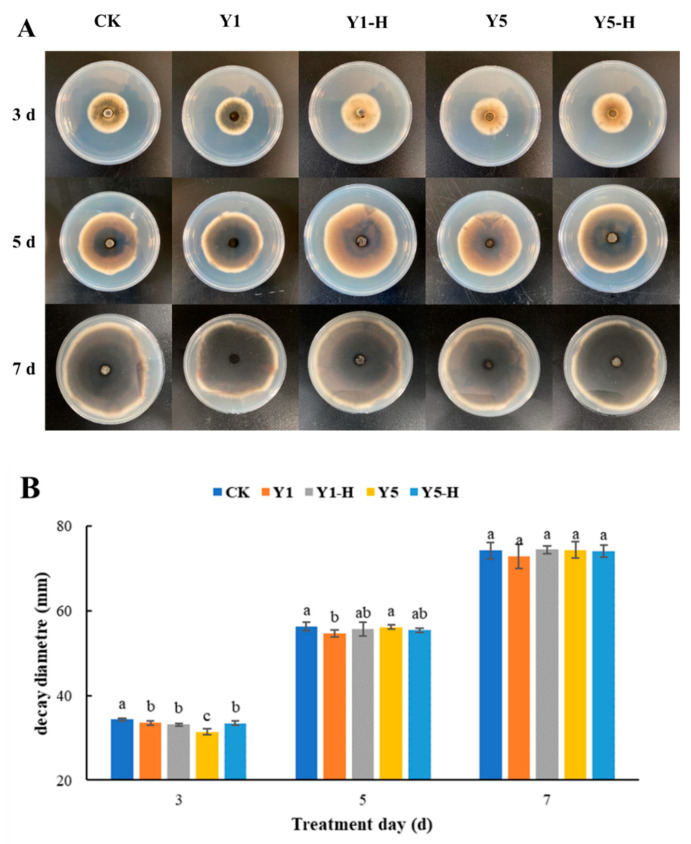
The effect of cell-free supernatant of *W. anomalus* on *A. alternata*. (**A**): A direct view of the shot; (**B**): The effect of cell-free supernatant of *W. anomalus* on the colony diameter of *A. alternata*. Different letters represented significant difference (*p* < 0.05).

As shown in Figure 1, after 3 days of co-culture, the cell-free supernatant of *W. anomalus* fermented for 5 days (Y5) exhibited specific inhibitory effects on the growth of *A. alternata* compared to the control group (34.3 mm) (*p* < 0.05). The mean colony diameter (31.4 mm) was lower than that of other treatment groups (33.6 mm, 33.2 mm, 33.4 mm, respectively). Following five days of co-culture, the cell-free supernatant fermented for 1 day (Y1) showed a certain inhibitory effect on *A. alternata* (*p* < 0.05). The mean colony diameter was 54.6 mm, which was less than the control group (56.3 mm), and the *W. anomalus* cell-free supernatant group fermented for 5 days (56.2 mm). After 7 days of co-culture, there was no apparent distinction in the colony diameters between the four treatment groups, and *A. alternata* continued to develop freely. As a result, *W. anomalus* cell-free filtrate showed a minimal inhibitory effect *on A. alternata*, and as the culture time increased, it exhibited no overall anti-*A. alternata* effects.

### 3.2. Effect of Volatile Metabolites of W. anomalus on the Growth of A. alternata

**Figure 2:**
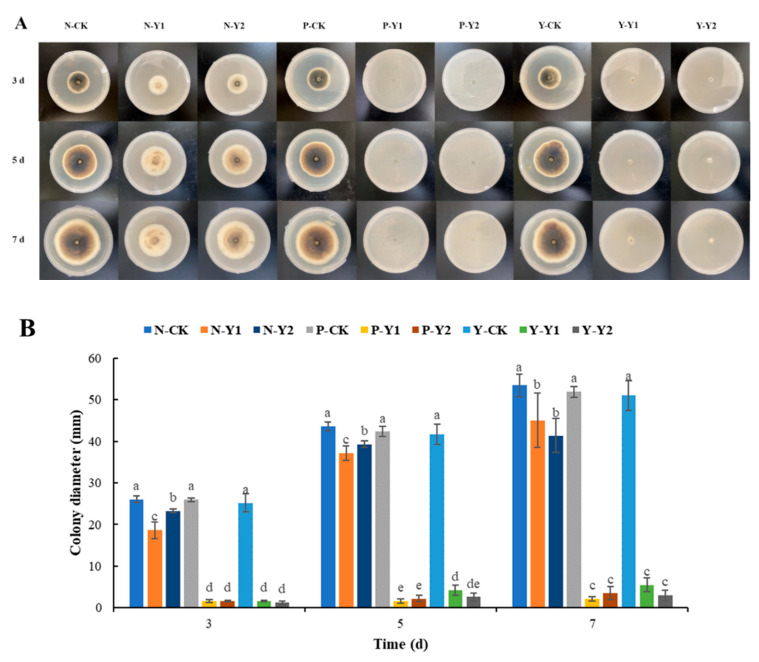
The effect of volatile substances of *W. anomalus* on *A. alternata*. (**A**): A direct view of the shot; (**B**): The effect of volatile metabolites of *W. anomalus* on the colony diameter of *A. alternata*. Different letters represented significant difference (*p* < 0.05).

The growth of *A. alternata* was significantly inhibited by the volatile metabolites of *W. anomalus* grown in several culture mediums (NYDA, PDA, and YPD), as illustrated in Figure 2. The study found that the inhibitory effect of volatile substances with *W. anomalus* cultured in NYDA for 1 day (N-Y1) was more significant than that with 2 days cultured (N-Y2) for *A. alternata* (*p* < 0.05). The colony diameters of N-Y1-treated *A. alternata* were 18.6 mm, 37.1 mm, and 45.0 mm after 3, 5, and 7 days, respectively. The colony diameters of N-Y2-treated *A. alternata* were 23.3 mm, 39.2 mm, and 41.4 mm after 3, 5, and 7 days, respectively. In comparison to the control group (26.0 mm, 43.6 mm, and 53.5 mm, respectively), the colony diameter of *A. alternata* was greatly decreased (*p* < 0.05) by volatile metabolites of *W. anomalus* cultivated in NYDA. Experimental data showed that on the 3rd, 5th, and 7th day, the inhibition rates of volatile substances of N-Y1 reached 23.9%, 13.3%, and 14.4%, respectively, which were significantly higher than N-Y2.

Similarly, the volatile metabolites of *W. anomalus* cultured in PDA and YPD medium showed a significant inhibitory effect on the growth of *A. alternata* under different culture times. The colony diameters of *A. alternata* exposed to volatile substances of *W. anomalus* cultured in PDA for 1 day (P-Y1) were 1.6 mm, 1.7 mm, and 2.1 mm after 3, 5, and 7 days, respectively. The growth diameters of *A. alternata* exposed to P-Y2 were 1.6 mm, 2.2 mm, and 3.5 mm after 3, 5, and 7 days, respectively. Compared with the control group, the colony diameter of *A. alternata* was significantly decreased (*p* < 0.05) in P-Y1 and P-Y2. The colony diameters of *A. alternata* exposed to volatile substances of *W. anomalus* cultured in YPD for 1 day (Y-Y1) were 1.7 mm, 4.2 mm, and 5.5 mm after 3, 5, and 7, respectively. The colony diameters of *A. alternata* exposed to Y-Y2 were 1.2 mm, 2.6 mm, and 3.0 mm after 3, 5, and 7 days, respectively. Compared with the control group (25.2 mm, 41.7 mm, 51.0 mm, respectively), the colony diameter of *A. alternata* was significantly decreased (*p* < 0.05) in Y-Y1 and Y-Y2. Experimental data showed that the highest inhibition rate (86.0%) was observed in volatile substances of *W. anomalus* cultured in PDA medium for 1 day (P-Y1) compared with other treatment groups; volatile substances under this condition had the most significant inhibition effect on *A. alternata*.

In conclusion, the volatile substances of *W. anomalus* cultured in PDA medium for 1 day significantly inhibited *A. alternata* growth. The volatile organic compounds of yeast were selected under this culture condition for detection and analysis in subsequent experiments.

### 3.3. Effect of Volatile Substances from W. anomalus on Tomato Black Spot Disease

**Figure 3:**
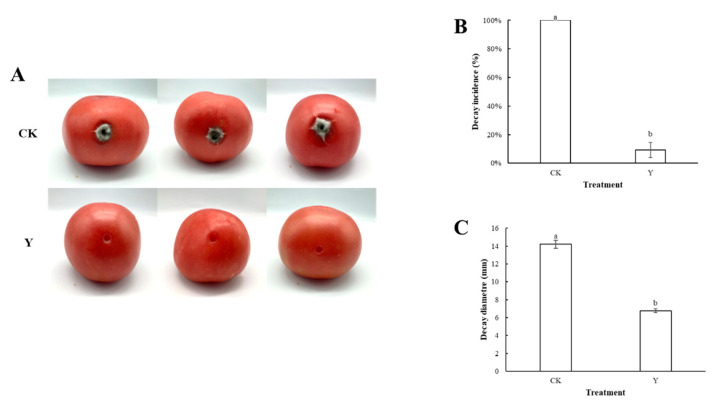
The effects of volatiles from *W. anomalus* on tomato black spot disease. (**A**): A direct view of tomatoes fumigated by *W. anomalus*; (**B**): The effects of *W. anomalus* fumigation on the rot rate of tomato black spots; (**C**): The effect of *W. anomalus* on the rot diameter of the tomato after fumigation. Different letters represented significant difference (*p* < 0.05).

After fumigation by *W. anomalus*, tomato postharvest black spot disease was suppressed, as Figure 3A illustrates. The tomatoes in the control group had a rot rate of 100%, while the tomatoes in the *W. anomalus* fumigation group had a rot rate of 9.3%, indicating a substantial inhibitory effect (*p* < 0.05), as demonstrated by Figure 3B,C. The rot diameter of tomatoes in the control group was 14.2 mm, and those in the *W. anomalus* fumigation group were 6.8 mm. It had an excellent inhibition effect on the spread of the lesion diameter (*p* < 0.05). Therefore, *W. anomalus* fumigation can significantly reduce the incidence of black spot disease and potentially control postharvest diseases.

### 3.4. W. anomalus VOCs Component Analysis (GC-MS)

**Figure 4:**
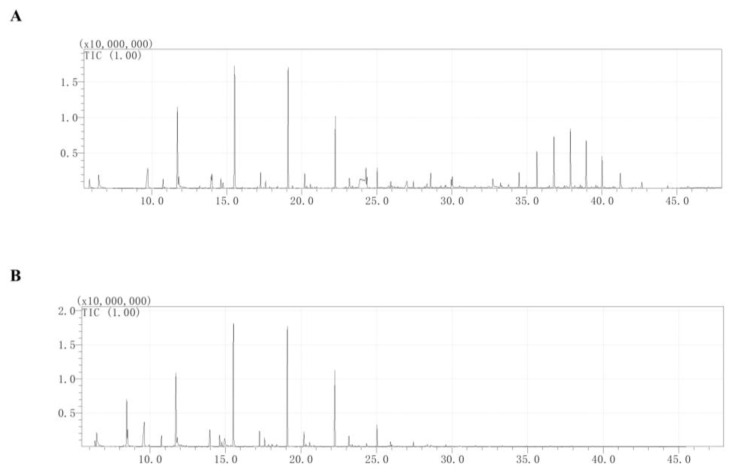
(**A**): GC-MS spectra of volatile organic compounds of CK; (**B**): GC-MS spectra of volatile organic compounds of *W. anomalus*.

Volatile substances were analyzed with GC-MS (Figure 4). The main volatile substances identified in *W. anomalus* were nine esters and three alcohol organic compounds (Table 1). Among them, the relative content of isoamyl acetate (1-Butanol, 3-methyl-, acetate) was the highest, accounting for 40.42%. The second substance was 2-butanol, 2-methyl-, acetate (15.61%). The third most common substance was phenylethyl alcohol (15.06%). The remaining components were acetic acid, butyl ester, hexanoic acid, ethyl ester, 3-(Methylthio)propyl acetate, 2,2,4-trimethyl-1,3-pentanediol diisobutyrate, acetic acid, 2-phenylethyl ester, 3-(Methylthio)-1-propanol, acetic acid, hexyl ester, 1-hexanol, 1,2-benzenedicarboxylic acid, and bis(2-methylpropyl) ester.

### 3.5. Impact of Isoamyl Acetate Fumigation on A. alternata In Vitro

**Figure 5:**
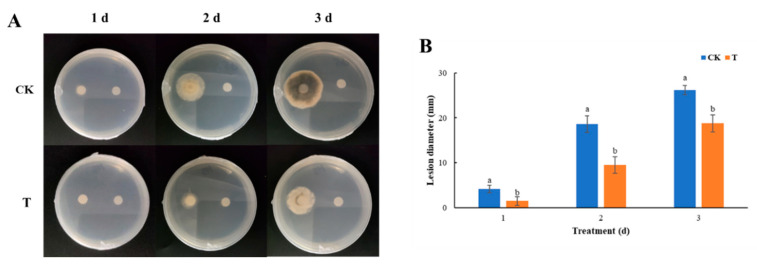
The inhibitory effect of isoamyl acetate on *A. alternata*. (**A**): A direct view of isoamyl acetate fumigation *A. alternata*; (**B**): The effect of isoamyl acetate fumigation on the colony diameter of *A. alternata*. Different letters represented significant difference (*p* < 0.05).

As shown in Figure 5A, fumigation with isoamyl acetate inhibited the mycelial growth of *A. alternata*, and its pigmentation was also inhibited. After fumigation with isoamyl acetate, the colony diameter of *A. alternata* was significantly decreased (*p* < 0.05), as demonstrated in Figure 5B. Within 1–3 days of isoamyl acetate fumigation, the growth diameters of *A. alternata* were 1.4 mm, 9.4 mm, and 18.7 mm, respectively, which were significantly lower than those of the control group (4.2 mm, 18.6 mm, and 26.2 mm, respectively) (*p* < 0.05). Hence, during the growth stages of *A. alternata*, isoamyl acetate exerts a notable inhibitory influence on its growth and development. The experimental findings indicate that isoamyl acetate constitutes the primary component responsible for the antifungal properties observed in the volatile metabolites of *W. anomalus*.

### 3.6. Effect of Fumigation with Isoamyl Acetate on Control of Postharvest Black Spot of Tomato

**Figure 6:**
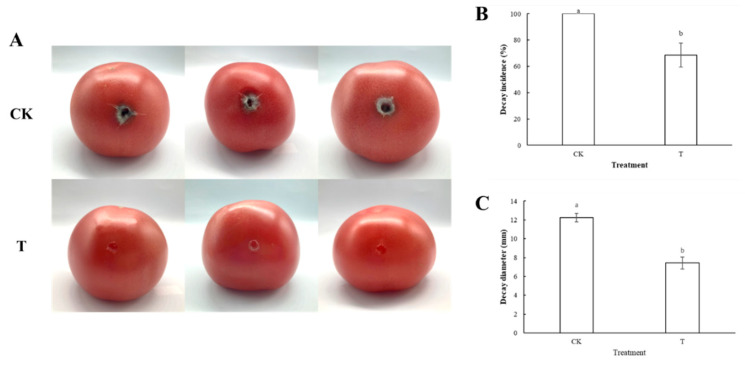
The effect of isoamyl acetate fumigation on tomato postharvest black spot disease. (**A**): A direct view of tomatoes fumigated with isoamyl acetate; (**B**): The effect of isoamyl acetate fumigation on the rot rate of tomato black spots; (**C**): The effect of *W. anomalus* on the rot diameter of the tomato after fumigation. Different letters represented significant difference (*p* < 0.05).

Isoamyl acetate fumigation has a specific prevention and control effect on postharvest black spots in tomatoes and inhibits the disease (Figure 6A). In the isoamyl acetate fumigation group, the tomatoes’ rot rate was 66.5% (Figure 6B), considerably lower than in the control group (100%), *p* < 0.05. As can be seen from Figure 6C, the fumigation group’s rot diameter was substantially less (*p* < 0.05) than that of the control group, which had a rot diameter of 12.2 mm. The fumigation group’s rot diameter was 7.4 mm. Therefore, isoamyl acetate fumigation can reduce the incidence of tomato black spots and inhibit the disease. The results of in vivo experiments suggested that isoamyl acetate was the main component of the antifungal activity of volatile metabolites of *W. anomalus*, and *W. anomalus* could be used as a biological fumigant to control postharvest diseases of fruits and vegetables.

## 4. Discussion

Antagonistic yeast metabolites play an important role in the prevention and control of postharvest diseases. In this experiment, the inhibitory effect of the nonvolatile substances and volatile substances of *W. anomalus* on *A. alternata* were studied. The results showed that the inhibitory effect of volatile substances was stronger than that of nonvolatile substances. The volatile substances that can counteract the antifungal properties of yeast are currently a hot research topic and are used to prevent and control plant diseases. The volatile metabolites of *Hanseniaspora uvarum* can reduce the natural decay of strawberries and have a control effect on gray mold in postharvest strawberries [17]. VOCs produced by *Candida sake* inhibit the growth of major pathogenic fungi in apples, including *P. expansum*, *B. Botrytis cinerea*, *A. alternata*, *Alternaria tenuissima*, and *Alternaria arborescens* [18]. Our current findings showed that among different metabolites of *W. anomalus*, its cell-free supernatant had no inhibitory effect on *A. alternata*. Still, its volatile substances could significantly inhibit the growth of *A. alternata*, which had the potential to prevent postharvest black spots in tomatoes. The volatile metabolites of *Candida pyralidae* and *Pichia kluyveri* in a cost-effective grape residue medium exhibited antifungal properties against postharvest *B. cinerea* and *Rhizopus stolonifera* [19]. The composition of volatile substances produced by yeast is highly influenced by its growth environment. For instance, different carbon and nitrogen sources in the medium can significantly alter the types of volatile compounds produced [20]. Previous experiments showed that *W. anomalus* is a strong aromatic yeast with a strong ester production capacity, and many of its volatile metabolites have certain floral and fruity aromas. This study showed that the volatile substances of *W. anomalus* had a strong inhibitory effect on *A. alternata* after 1 day of cultivation in PDA medium.

The results showed that the volatile substances of *W. anomalus* on PDA were mainly esters and alcohols, including isoamyl acetate, 2-methylbutyl acetate, phenyl ethanol, butyl acetate, ethyl n-caproate, and other major components. Isoamyl acetate is the main organic compound in the volatile compounds of *W. anomalus*, accounting for 40.4%. It has been found that isoamyl acetate is one of the volatile metabolites of some fruits and vegetables, such as bananas [21], peaches [22], yeast (ascomycetes yeasts [23]), and bacillus, and isoamyl acetate has been confirmed to have antibacterial activity. The isoamyl acetate was the primary volatile substance in *Bacillus subtilis* Y8, and isoamyl acetate inhibited the germination of conidia of *Curvularia lunata* and increased the accumulation of reactive oxygen species in pathogenic bacteria [24]. It also has a control effect on leaf spot disease in maize. The isoamyl acetate had an inhibitory effect on *A. alternata* both in vitro and in vivo, delaying black spots in cherry tomatoes [25]. Through in vivo and in vitro experiments, it was found that isoamyl acetate had an inhibitory effect on the growth of *A. alternata* and had a certain control effect on postharvest black spots in tomatoes.

While our study highlights the potential of isoamyl acetate as an antifungal agent against *A. alternata*, using a single strain limits the generalizability of the findings. Sensitivity to antifungal compounds can vary considerably within fungal populations. Future investigations employing a more comprehensive range of *A. alternata* strains would be crucial to assess the broader applicability of isoamyl acetate for *A. alternata* control. Additionally, quantifying the specific volatile metabolites produced by *W. anomalus* that contribute to *A. alternata* growth inhibition would provide valuable insights into the underlying mechanisms. Such knowledge would be essential for optimizing the application of isoamyl acetate to prevent and treat tomato black spots caused by *A. alternata*.

## 5. Conclusions

In this study, we analyzed the the prevention and control effect of metabolites of *W. anomalus* on tomato black spot disease, and the inhibition effect of main components of *W. anomalus* on *A. alternata* was investigated. The results showed that *W. anomalus* volatile substances showed effects on the postharvest black spots in tomatoes. The analysis showed that isoamyl acetate was the primary active substance, which could potentially control the postharvest black spots in tomatoes. *W. anomalus* volatile substances can be applied to biological fumigants and are expected to be a future alternative to chemical fungicides.

## Figures and Tables

**Table 1:** Detection of VOCs of *W. anomalus* in PDA medium.

Volatile Compounds	CAS	Chemical Formula	Relative Peak Area Ratio (%)	Odour
1-Butanol, 3-methyl-, acetate	123-92-2	C_7_H_14_O_2_	40.42	The smell of bananas
1-Butanol, 2-methyl-, acetate	624-41-9	C_7_H_14_O_2_	15.61	The smell of bananas
Phenylethyl Alcohol	60-12-8	C_8_H_10_O	15.06	A sweet rose-like fragrance
Acetic acid, butyl ester	123-86-4	C_6_H_12_O_2_	5.17	-
Hexanoic acid, ethyl ester	123-66-0	C_8_H_16_O_2_	3.04	Pineapple fruit aroma
3-(Methylthio)propyl acetate	16630-55-0	C_6_H_12_O_2_S	2.96	-
2,2,4-Trimethyl-1,3-pentanediol diisobutyrate	6846-50-0	C_16_H_30_O_4_	2.29	-
Acetic acid, 2-phenylethyl ester	103-45-7	C_10_H_12_O_2_	2.19	A honey-like aroma with a pink aroma, similar to an apple-like fruit aroma, and with a cocoa and whiskey-like aroma.
1-Propanol, 3-(methylthio)-	505-10-2	C_4_H_10_OS	2.05	Strong smell of onion and meat.
Acetic acid, hexyl ester	142-92-7	C_8_H_16_O_2_	1.57	Rich fruity smell
1-Hexanol	111-27-3	C_6_H_14_O	1.42	Light green twigs and leaves smell, with wine, fruit, and fat smell
1,2-Benzenedicarboxylic acid, bis(2-methylpropyl) ester	84-69-5	C_16_H_22_O_4_	1.27	A slightly aromatic scent

## Data Availability

The original contributions presented in the study are included in the article/Supplementary Materials, further inquiries can be directed to the corresponding author.

## Supplementary Materials

The following supporting information can be downloaded at: https://www.mdpi.com/article/10.3390/foods13121949/s1, Table S1: Control and treatment groups used for assessing of by nonvolatile metabolites of *W. anomalus* on *A. alternata*; Table S2: Control and treatment groups used for assessing of by volatile metabolites of *W. anomalus* on *A. alternata*; Table S3: Control and treatment groups used for assessing of by volatile metabolites of *W. anomalus* on tomato black spot disease; Table S4: Control and treatment groups used for assessing of by isoamyl acetate fumigation on *A. alternata in vitro*; Table S5: Control and treatment groups used for assessing of by isoamyl acetate fumigation on tomato black spot disease.
