# Supplementary material for: Volatile Organic Compounds of Wickerhamomyces anomalus Prevent Postharvest Black Spot Disease in Tomato
Source: Foods. 2024 Jun 20;13(12):1949. doi: 10.3390/foods13121949 (PMC11202777; doi:10.3390/foods13121949)
Supplement: Supplementary file 1 [file foods-13-01949-s001.zip › foods-3032827-supplementary.pdf]

Table S1. Control and treatment groups used for assessing of by nonvolatile metabolites of *W. anomalus* on *A. alternata*

Group	Treatment
CK	treated with sterile water
Y1	treated with cell-free supernatant after 1 day of culture
Y1-H	treated with heat killed solution after 1 day of culture
Y5	treated with cell-free supernatant after 5 days of culture
Y5-H	treated with heat killed solution after 5 days of culture

Table S2. Control and treatment groups used for assessing of by volatile metabolites of *W. anomalus* on *A. alternata*

Group	Treatment
N-CK	Normal saline in NYDB medium
N-Y1	*W. anomalus* cultured in NYDB medium for 1 day
N-Y2	*W. anomalus* cultured in NYDB medium for 2 days
P-CK	Normal saline in PDA medium
P-Y1	*W. anomalus* cultured in PDA medium for 1 day
P-Y2	*W. anomalus* cultured in PDA medium for 2 days
Y-CK	Normal saline in YPD medium
Y-Y1	*W. anomalus* cultured in YPD medium for 1 day
Y-Y2	*W. anomalus* cultured in YPD medium for 2 days

Table S3. Control and treatment groups used for assessing of by volatile metabolites of *W. anomalus* on tomato black spot disease

Group	Treatment
CK	Normal saline treatment group
Y	*W. anomalus* fumigation group

Table S4. Control and treatment groups used for assessing of by isoamyl acetate fumigation on *A. alternata in vitro*

Group	Treatment
CK	No fumigation
T	Isoamyl acetate treatment

Table S5. Control and treatment groups used for assessing of by isoamyl acetate fumigation on tomato black spot disease

Group	Treatment
CK	No fumigation
T	Isoamyl acetate treatment
